# Supplementary material for: Proteome allocation is linked to transcriptional regulation through a modularized transcriptome
Source: Nat Commun. 2024 Jun 19;15:5234. doi: 10.1038/s41467-024-49231-y (PMC11187210; doi:10.1038/s41467-024-49231-y)
Supplement: Supplementary file 1 — Supplementary Information [file 41467_2024_49231_MOESM1_ESM.pdf]

Supplementary Information

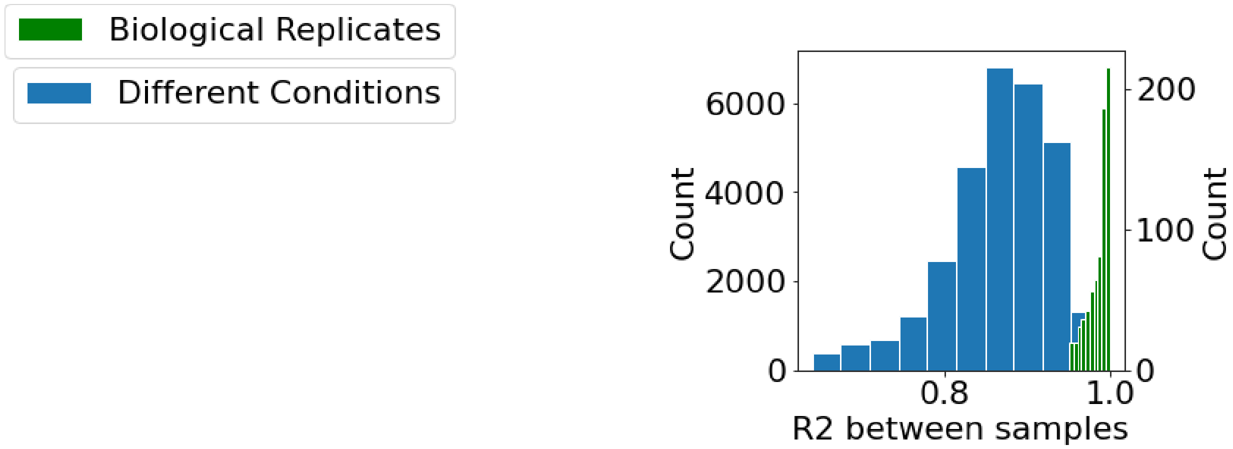

Supplementary Figure 1. PRECISE1k replicate correlations. R^2 values between biological replicates and random samples within PRECISE1k.

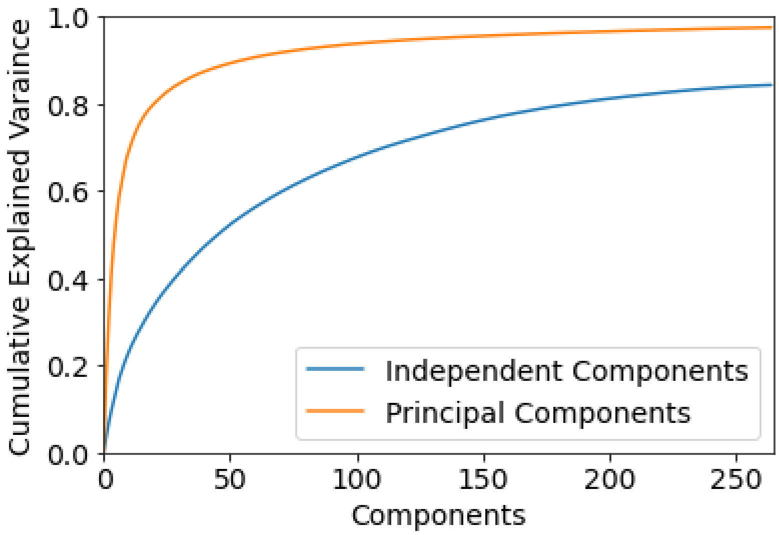

Supplementary Figure 2. PRECISE1k explained variance. Explained variance plot for PRECISE1k and its components.

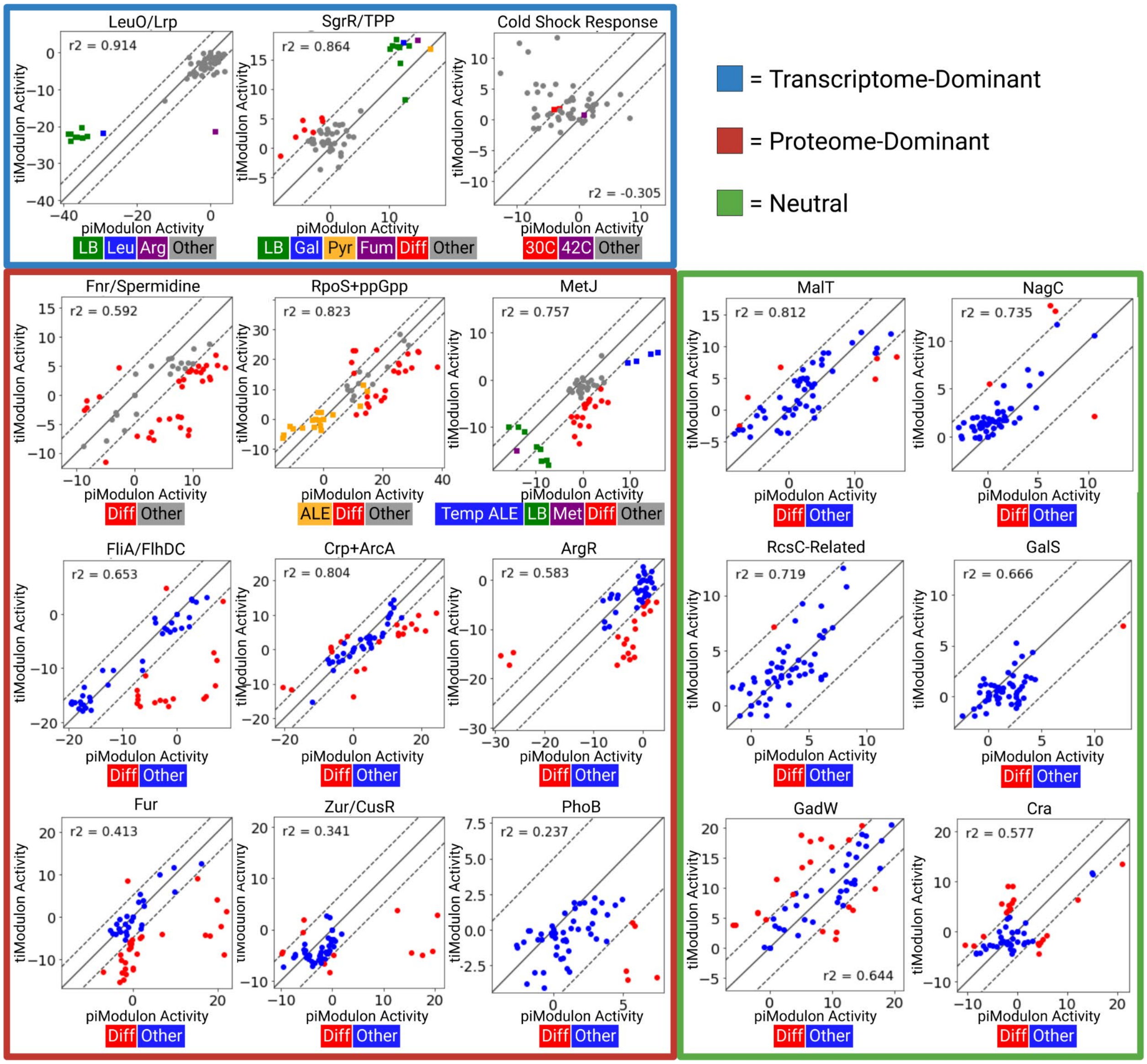

Supplementary Figure 3. Comparing piModulon and tiModulon activities for all matched pairs. Differential iModulon Activity (DiMA) plots for all matched iModulons between the two datasets. iModulons that are transcriptome-dominant (signal more active in the tiModulon than the piModulon) are highlighted in blue. iModulons that are proteome-dominant are highlighted in red, and iModulons that are neutral are highlighted in green. Activities are considered differentially activated for samples that lie outside the significance threshold (dashed line). Legends for each plot are placed below each plot. *Abbreviations LB: Lysogeny Broth, Leu: Leucine Supplement, Arg: Arginine Supplement, Gal: Galactose Carbon Source, Pyr: Pyruvate Carbon Source, Fum: Fumerate Carbon Source, Diff: Differentially Activated, Temp: Temperature, Met: Methionine Supplement. n=57 conditions.*

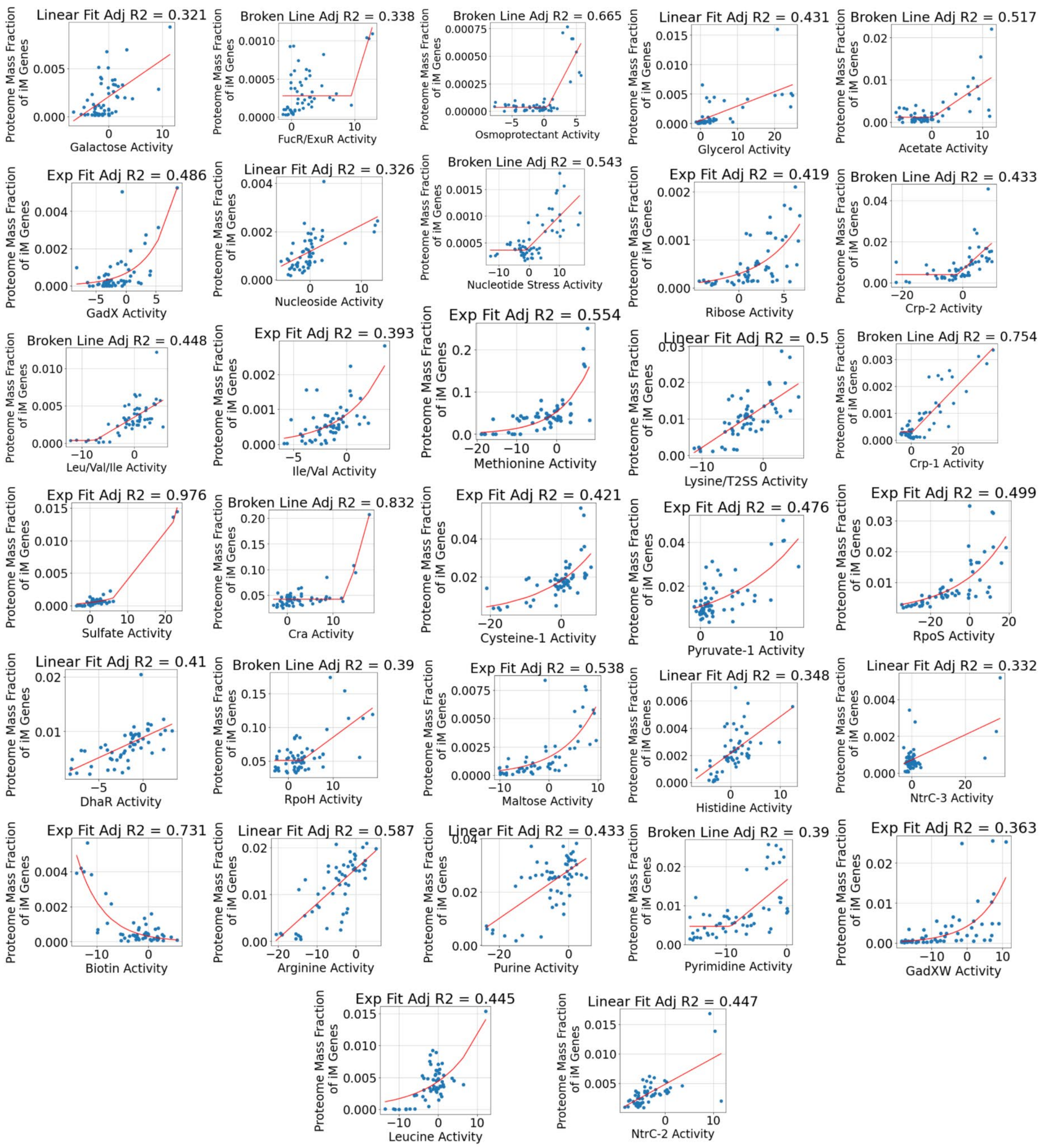

Supplementary Figure 4. Scatter plots and regressions for all tiModulons with strong correlations. Scatter plots for all tiModulon activities and their measured proteome mass fraction of the associated genes for tiModulons with strong correlations. tiModulons are charaterized based on which regression method (linear, exponential, and broken line) resulted in the best adjusted R^2 value. The regression method and R^2 value can be found above each plot, with the tiModulon name below. Individual samples are scatter plotted in blue, and the regression line is in red. n=57 conditions.

A

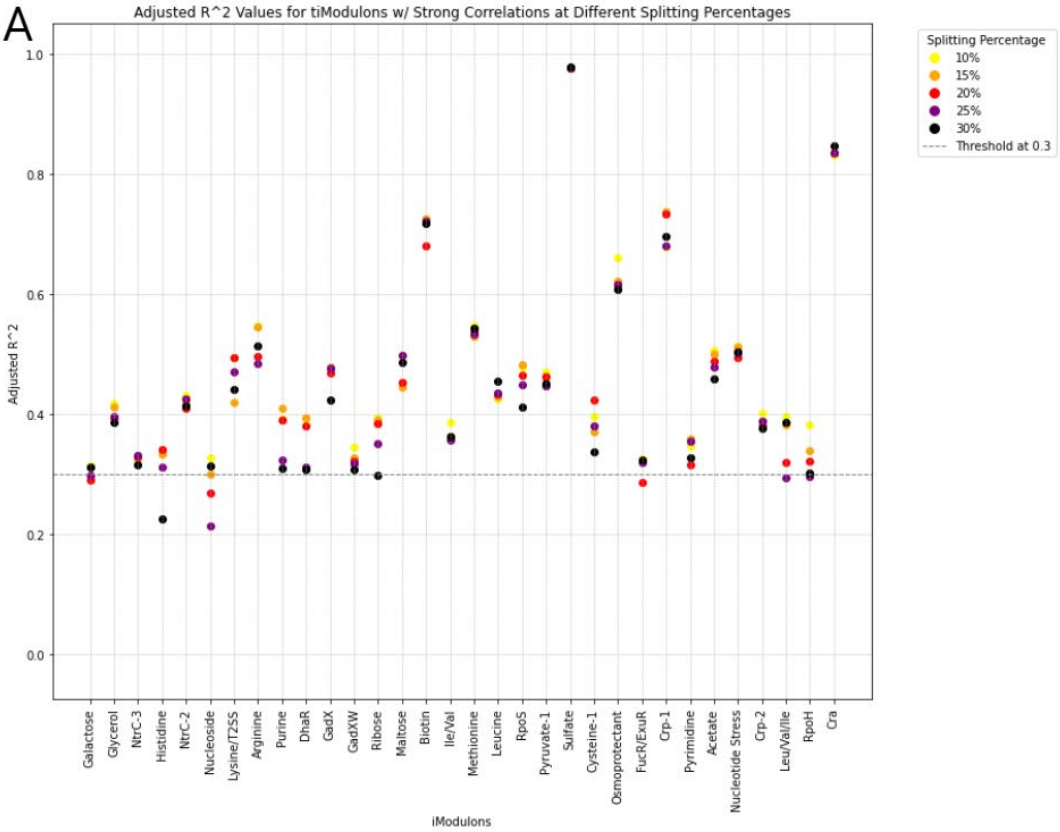
Adjusted R^2 Values for tiModulons w/ Strong Correlations at Different Splitting Percentages
Adjusted R^2
iModulons
1.0
0.8
0.6
0.4
0.2
0.0
Splitting Percentage
10%
15%
20%
25%
30%
Threshold at 0.3
Galactose
Glycerol
NtrC-3
Histidine
NtrC-2
Nucleoside
Lysine/T2SS
Arginine
Purine
DhaR
GadX
GadXW
Ribose
Maltose
Biotin
Ile/Val
Methionine
Leucine
RpoS
Pyruvate-1
Sulfate
Cysteine-1
Osmoprotectant
FucR/ExuR
Crp-1
Pyrimidine
Acetate
Nucleotide Stress
Crp-2
Leu/Val/Ile
RpoH
Cra

B

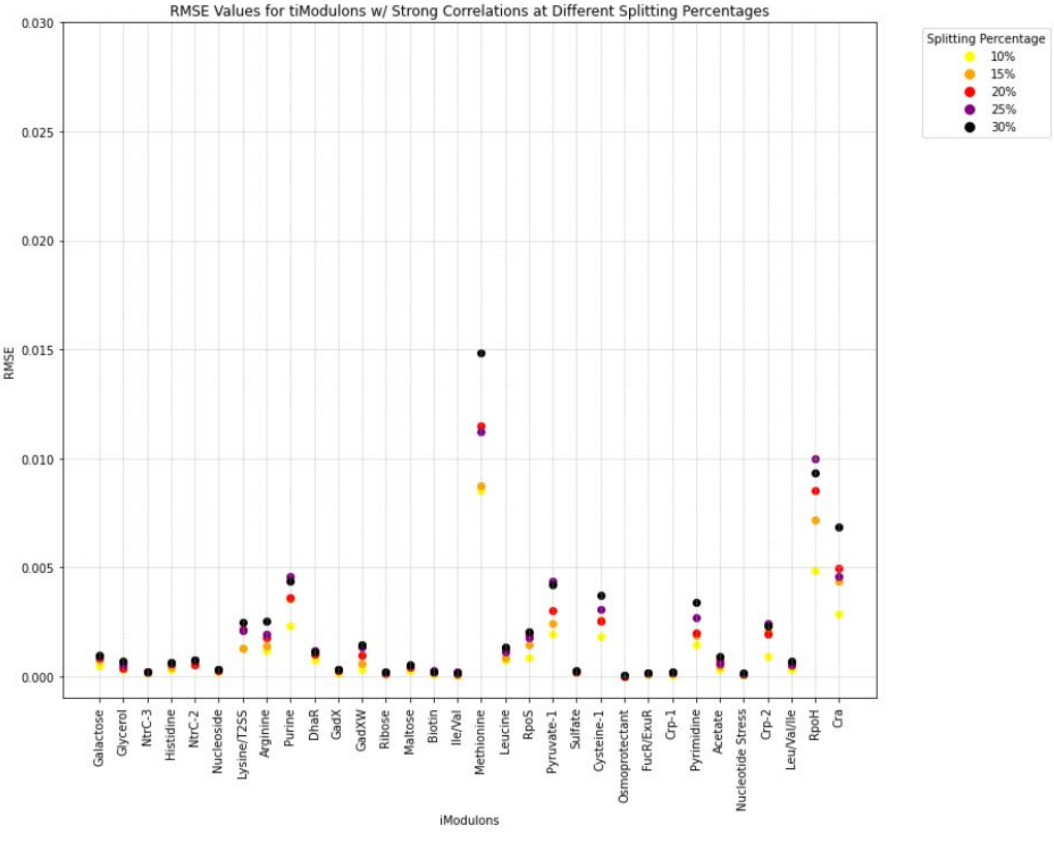
RMSE Values for tiModulons w/ Strong Correlations at Different Splitting Percentages
RMSE
iModulons
0.030
0.025
0.020
0.015
0.010
0.005
0.000
Splitting Percentage
10%
15%
20%
25%
30%
Galactose
Glycerol
NtrC-3
Histidine
NtrC-2
Nucleoside
Lysine/T2SS
Arginine
Purine
DhaR
GadX
GadXW
Ribose
Maltose
Biotin
Ile/Val
Methionine
Leucine
RpoS
Pyruvate-1
Sulfate
Cysteine-1
Osmoprotectant
FucR/ExuR
Crp-1
Pyrimidine
Acetate
Nucleotide Stress
Crp-2
Leu/Val/Ile
RpoH
Cra

Supplementary Figure 5. Leave-one-out cross-validation with additional holdouts for tiModulons w/ Strong Correlations. A) Adjusted R2 values for each regression model at various splitting percentages, only for tiModulons w/ strong correlations to proteome allocation. B) Root Mean Square Errors for each regression model at various splitting percentages, only for tiModulons w/ strong correlations to proteome allocation.

Discussion of Supplementary Figure 5:
The regression models for iModulons that are strongly correlated with their proteome allocation are actually fairly robust even for 57 samples. The Adjusted R2 values stay above the 0.3 threshold for almost all splitting percentages across iModulons except for a couple of extreme (>20%) splits (**Figure S5A**). This indicates that the models fit the data well. Furthermore, the root mean square errors (RMSEs) for the strongly correlated iModulons are also tight with an average standard deviation of 0.000438 (**Figure S5B**). This average standard deviation indicates that the model can accurately infer the proteome allocation for new data points. The scale of RMSE relates to the magnitude of proteome allocation, which is why the Cra, Methionine, and RpoH iModulons all have relatively high RMSEs (0.043367, 0.043133, and 0.033590 % of the proteome, respectively). These three iModulons are the three largest by proteome mass (see **Figure 5D** in main text) and thus vary the most, so seeing them stand out is expected.

A

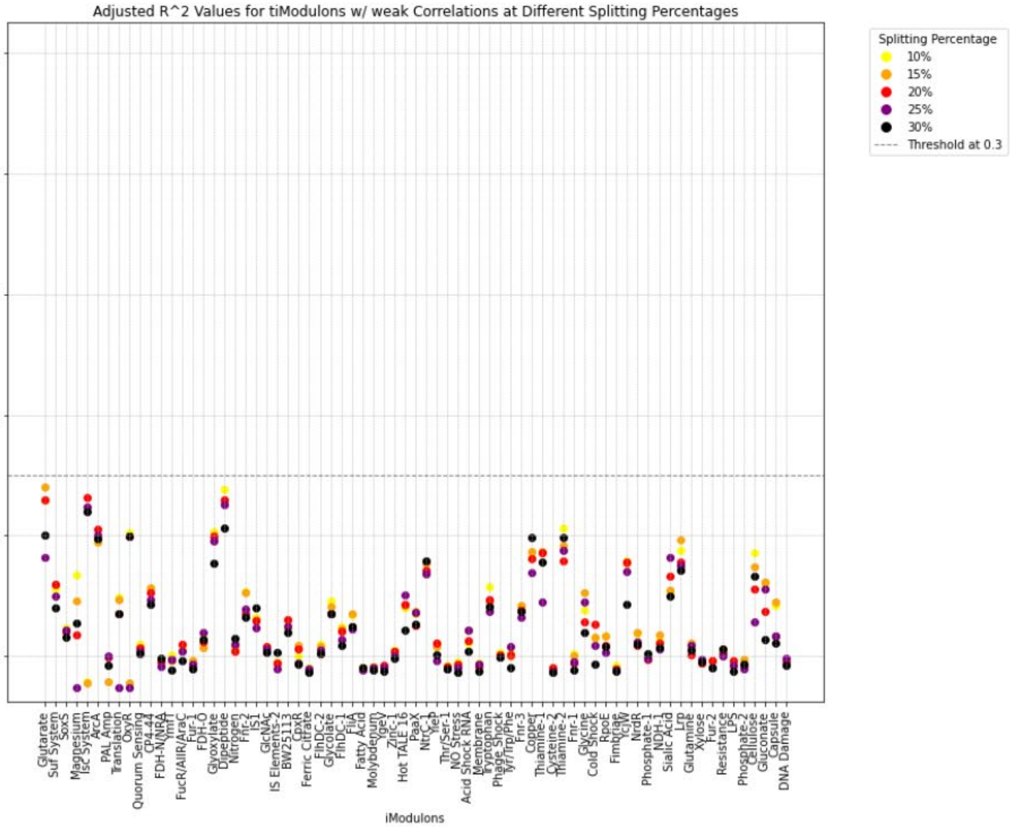
Adjusted R^2 Values for tiModulons w/ weak Correlations at Different Splitting Percentages
Adjusted R^2
1.0
0.8
0.6
0.4
0.2
0.0
iModulons
Glutarate
Suf System
SoxS
Magnesium
Isc System
ArcA
PAL Amp
Translation
OxyR
Quorum Sensing
CP4-44
FDH-N/NRA
YmfT
FucR/AllR/AraC
Fur-1
FDH-O
Glyoxylate
Dipeptide
Nitrogen
Fnr-2
IS1
GlcNAc
IS Elements-2
BW25113
CpxR
Ferric Citrate
FlhDC-2
Glycolate
FlhDC-1
FliA
Fatty Acid
Molybdenum
YgeV
Zinc-1
Hot TALE 16
PaaX
NtrC-1
YieP
Thr/Ser-1
NO Stress
Acid Shock RNA
Membrane
Tryptophan
Phage Shock
Tyr/Trp/Phe
Fnr-3
Copper
Thiamine-1
Cysteine-2
Thiamine-2
Fnr-1
Glycine
Cold Shock
RpoE
Fimbriae
YcjW
NrdR
Phosphate-1
NDH-1
Sialic Acid
Lrp
Glutamine
Xylose
Fur-2
Resistance
LPS
Phosphate-2
Cellulose
Gluconate
Capsule
DNA Damage
Splitting Percentage
10%
15%
20%
25%
30%
Threshold at 0.3

B

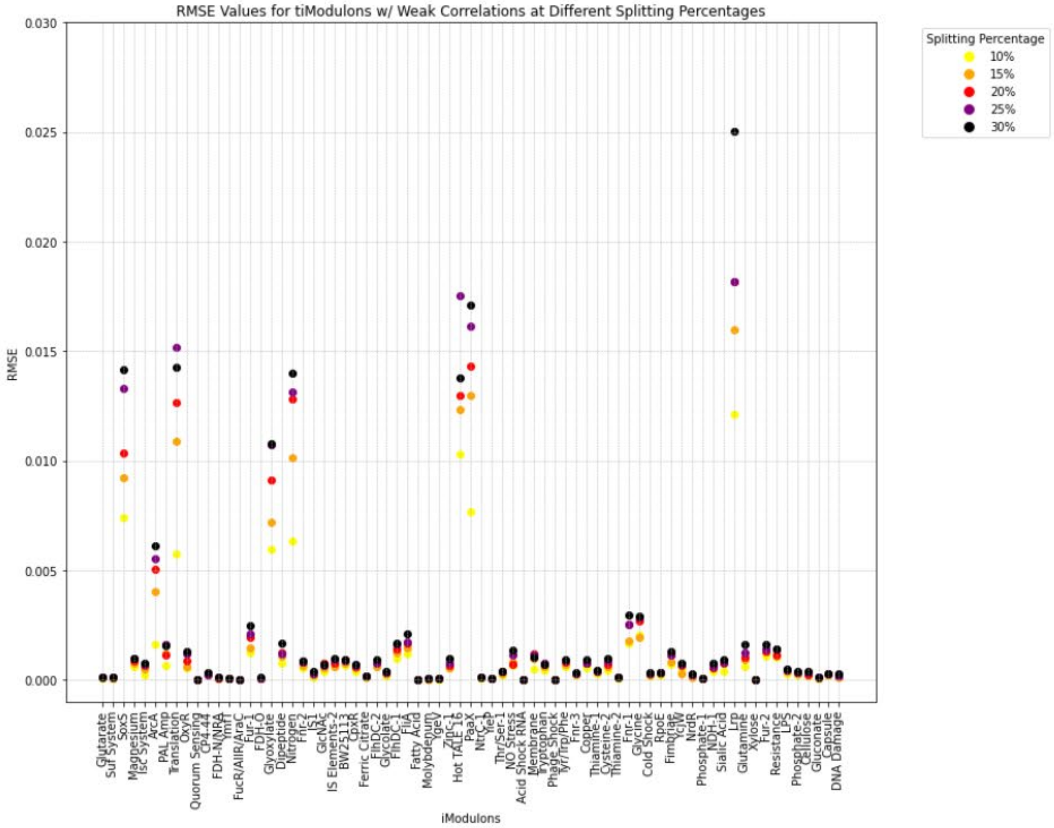
RMSE Values for tiModulons w/ Weak Correlations at Different Splitting Percentages
RMSE
0.030
0.025
0.020
0.015
0.010
0.005
0.000
iModulons
Glutarate
Suf System
SoxS
Magnesium
Isc System
ArcA
PAL Amp
Translation
OxyR
Quorum Sensing
CP4-44
FDH-N/NRA
YmfT
FucR/AllR/AraC
Fur-1
FDH-O
Glyoxylate
Dipeptide
Nitrogen
Fnr-2
IS1
GlcNAc
IS Elements-2
BW25113
CpxR
Ferric Citrate
FlhDC-2
Glycolate
FlhDC-1
FliA
Fatty Acid
Molybdenum
YgeV
Zinc-1
Hot TALE 16
PaaX
NtrC-1
YieP
Thr/Ser-1
NO Stress
Acid Shock RNA
Membrane
Tryptophan
Phage Shock
Tyr/Trp/Phe
Fnr-3
Copper
Thiamine-1
Cysteine-2
Thiamine-2
Fnr-1
Glycine
Cold Shock
RpoE
Fimbriae
YcjW
NrdR
Phosphate-1
NDH-1
Sialic Acid
Lrp
Glutamine
Xylose
Fur-2
Resistance
LPS
Phosphate-2
Cellulose
Gluconate
Capsule
DNA Damage
Splitting Percentage
10%
15%
20%
25%
30%

Supplementary Figure 6. Leave-one-out cross-validation with additional holdouts for tiModulons w/ Weak Correlations. A) Adjusted R2 values for each regression model at various splitting percentages, only for tiModulons w/ weak correlations to proteome allocation. B) Root Mean Square Errors for each regression model at various splitting percentages, only for tiModulons w/ weak correlations to proteome allocation.

Discussion of Supplementary Figure 6:
The Adjusted R2 values consistently stay below 0.3, indicating that the models don't fit the data well (**Figure S6A**). The average standard deviation for the RMSEs for the weakly correlated iModulons is 0.000465, which is more than the average standard deviation for strongly correlated iModulons (**Figure S6B**). These iModulons are much smaller by proteome mass, so one would expect the standard deviation to be less since it should scale with the magnitude of proteome allocation (only Translation, Lrp, ArcA, and Glyoxylate are large by mass, but we unexpectedly also see other iModulons with high RMSEs, see **Figure 5D** in main text). These results indicate that not only do the models not fit the data well, they also don't predict new data well.

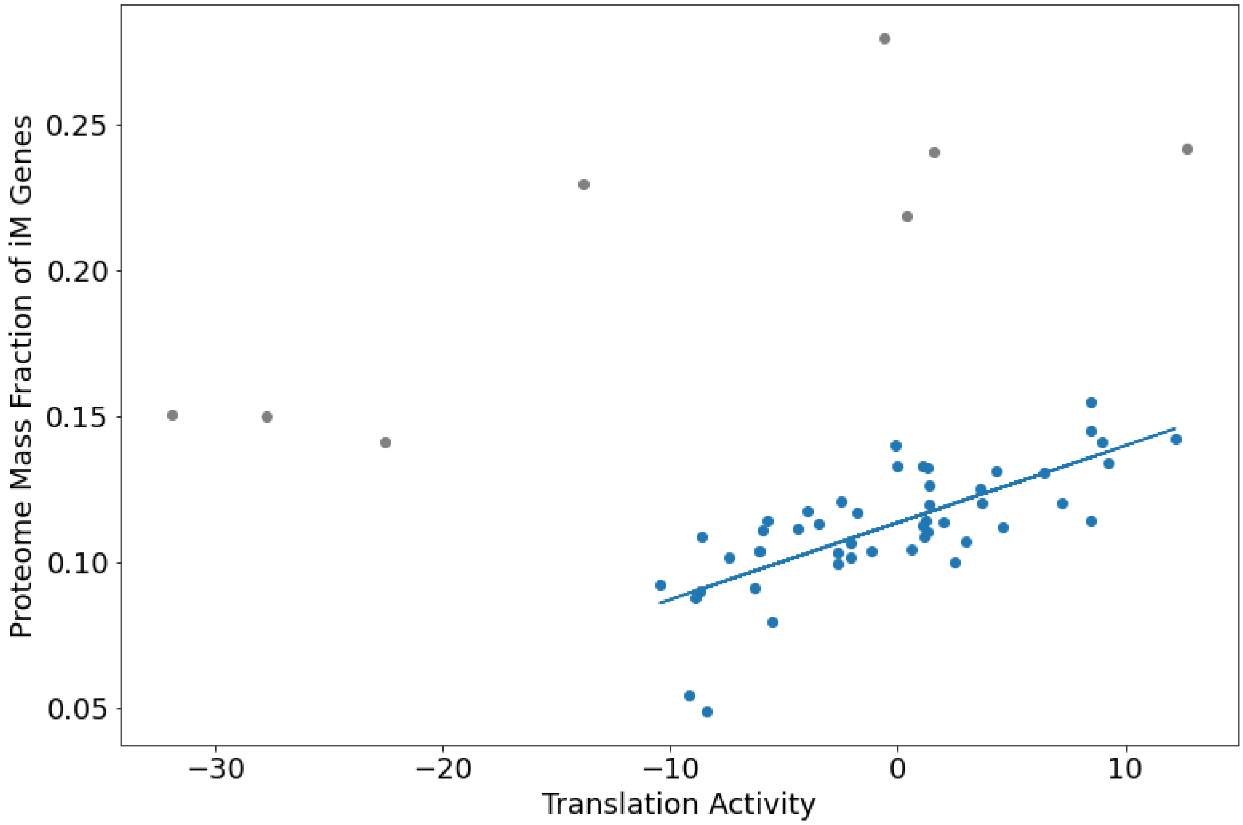

Supplementary Figure 7. Proteome allocation of the translation tiModulon. PRECISE1k Translation tiModulon activities plotted against their associated proteome mass fractions. There is a strong correlation between samples except for a few outliers (gray). R^2 = 0.737 without outliers, R^2 = 0.135 with outliers. n=57 conditions.

Supplementary Table 1. Matched tiModulons and piModulons

piModulon	tiModulon	Correlation
MalT	Maltose	0.517757
RcsC-related	UC-4	0.503688
RcsC-related	pts ALE	0.505555
RpoS+ppGpp	RpoS	0.63325
NagC	GlcNAc	0.656724
FliA/FlhDC	FlhDC-2	0.336105
FliA/FlhDC	FliA	0.792489
Fur	Fur-1	0.68668
GalS	Galactose	0.535344
ArgR	Arginine	0.332504
Cold Shock Response	Cold Shock	0.489819
SgrR/Thiamine diphosphate	Thiamine-2	0.260406
SgrR/Thiamine diphosphate	Thiamine-1	0.65899
PhoB	Phosphate-1	0.427802
PhoB	tpiA KO	0.332344
Zur/CusR	Zinc-1	0.411696
Zur/CusR	Copper	0.2603
GadW	GadXW	0.581244
Translation-Null	Translation	0.391143
Fnr/Spermidine	Fnr-1	0.347905
Fnr/Spermidine	Fnr-3	0.401703
LeuO/Lrp	Lrp	0.65983

LeuO/Lrp	Pyruvate-1	0.264165
Crp+ArcA	Crp-2	0.532799
Crp+ArcA	Maltose	0.255092
Cra	Cra	0.680723
MetJ	Methionine	0.448365
Fimbriae	Fimbriae	0.471338
