# Supplementary material for: Proteome allocation is linked to transcriptional regulation through a modularized transcriptome
Source: Nat Commun. 2024 Jun 19;15:5234. doi: 10.1038/s41467-024-49231-y (PMC11187210; doi:10.1038/s41467-024-49231-y)
Supplement: Supplementary file 3 — Description of Additional Supplementary Files [file 41467_2024_49231_MOESM3_ESM.pdf]

Description of Additional Supplementary Files

Supplementary Information – Extra Figures and Tables

Supplementary Data 1 – Protein Mass Fractions

Supplementary Data 2 – ProteomICA decomposition matrices, iModulons, and sample tables

Supplementary Data 3 – PRECISE1K Subset decomposition matrices

Supplementary Data 4 – Matched sample table
